# Multi omics analysis of mitophagy subtypes and integration of machine learning for predicting immunotherapy responses in head and neck squamous cell carcinoma

**DOI:** 10.18632/aging.205964

**Published:** 2024-06-21

**Authors:** Junzhi Liu, Huimin Li, Qiuping Dong, Zheng Liang

**Affiliations:** 1 Department of Otorhinolaryngology, Tianjin Medical University General Hospital, Tianjin 300052, China; 2 Laboratory of Cancer Cell Biology, National Clinical Research Center for Cancer, Key Laboratory of Cancer Immunology and Biotherapy, Tianjin’s Clinical Research Center for Cancer, Tianjin Medical University Cancer Institute and Hospital, Tianjin, China

**Keywords:** mitophagy, single cell, machine learning, immunotherapy response, immune microenvironment

## Abstract

Mitophagy serves as a critical mechanism for tumor cell death, significantly impacting the progression of tumors and their treatment approaches. There are significant challenges in treating patients with head and neck squamous cell carcinoma, underscoring the importance of identifying new targets for therapy. The function of mitophagy in head and neck squamous carcinoma remains uncertain, thus investigating its impact on patient outcomes and immunotherapeutic responses is especially crucial. We initially analyzed the differential expression, prognostic value, intergene correlations, copy number variations, and mutation frequencies of mitophagy-related genes at the pan-cancer level. Through unsupervised clustering, we divided head and neck squamous carcinoma into three subtypes with distinct prognoses, identified the signaling pathway features of each subtype using ssGSEA, and characterized subtype B as having features of an immune desert using various immune infiltration calculation methods. Using multi-omics data, we identified the genomic variation characteristics, mutated gene pathway features, and drug sensitivity features of the mitophagy subtypes. Utilizing a combination of 10 machine learning algorithms, we have developed a prognostic scoring model called Mitophagy Subgroup Risk Score (MSRS), which is used to predict patient survival and the response to immune checkpoint blockade therapy. Simultaneously, we applied MSRS to single-cell analysis to explore intercellular communication. Through laboratory experiments, we validated the biological function of SLC26A9, one of the genes in the risk model. In summary, we have explored the significant role of mitophagy in head and neck tumors through multi-omics data, providing new directions for clinical treatment.

## INTRODUCTION

HNSCC, also known as head and neck squamous cell carcinoma, accounts for 95% of cancerous growth in the head and neck area, resulting in over 316,000 deaths worldwide annually [1, 2]. Similar to other forms of solid cancers, HNSCC encounters various obstacles during its progression, such as oxygen deprivation, inadequate nourishment, immune cell toxicity, and diverse treatment modalities [3, 4]. Maintaining a functional network of mitochondria in tumors relies heavily on the process of mitophagy, which is responsible for selectively eliminating malfunctioning mitochondria [5–7]. The resultant decomposition byproducts can function as bioenergetic intermediaries to facilitate unimpeded expansion. In HNSCC, targeting mitophagy in different signaling pathways has been found to prevent oncogenesis [8–10]. Hence, it is beneficial to investigate the importance of mitophagy in HNSCC, as it aids in comprehending the processes of tumor development and aiming at malignant proliferation.

Mitophagy, a selective form of autophagy confined to the mitochondria, targets the elimination of damaged and senescent mitochondria, significantly impacting the maintenance of cellular mitochondrial quantity and quality [11, 12]. This process is crucial in regulating the balance of cancer cells, where it can function to promote or suppress cancerous growth. Although mitophagy acts as a defense against various environmental insults by preserving mitochondrial integrity and energy homeostasis, its dysregulation can contribute to mitochondrial network abnormalities and energy production disruptions, influencing cancer development and progression [13, 14]. The activation of mitophagy can vary with cell types, involving different pathways like PINK1/Parkin, BNIP3/Nix, and FUNDC1 [11]. In cancer cells, mitophagy has a dual effect, serving as a tumor promoter or inhibitor. Initially, by clearing defective mitochondria, it protects cells from oxidative and DNA damage, potentially preventing tumor initiation. However, in established tumors, especially those that are advanced or aggressive, cancer cells harness mitophagy to alleviate oxidative stress and recycle components for growth and survival [15–17]. Mitophagy’s connection with cancer cell metabolic reprogramming, drug resistance, and stem-like properties is well-established. Key pathways, including the classic PINK1-Parkin and the hypoxia-driven BNIP3/Nix and FUNDC1 pathways, are implicated in the metabolic reconfiguration of cancer cells, particularly affecting glycolysis. In the absence of Parkin, PTEN degradation occurs, modulating glycolysis and activating PI3K/AKT signaling through Parkin-PTEN interactions [18]. Furthermore, the PINK1-Parkin pathway can trigger a HIF1α-dependent Warburg effect, which is a hallmark of cancer metabolism, by degrading mitochondrial transporters SLC25A37 and SLC25A28. This leads to an accumulation of mitochondrial iron, supporting the relentless division and growth of cancer cells [19].

The development of cancer is frequently associated with the initiation of oncogenic signals and an adjustment to low oxygen environments. It is observed that Parkin inhibits glycolysis by binding with pyruvate kinase M2 (PKM2) and facilitating the ubiquitination and subsequent breakdown of HIF1a. This action hampers the stimulation of proteins that promote glycolysis and various transcriptional targets [20]. In a similar vein, the lack of PINK1 correlates with the Warburg effect, which is marked by the stabilization of HIF1a and a reduction in PKM2 efficacy [21]. Disruption of essential elements of the mitophagy pathway can result in its dysfunction. Conversely, a rise in mitochondrial ROS is documented to transcriptionally heighten HIF1a, triggering the metabolism of glycolysis and the expression of its associated genes (BNIP3, NIX, FUNDC1), thereby influencing mitophagy [22]. Research indicates that mitophagy plays a crucial role in sustaining stem cell reserves, including those traits that are stem-like within cancer stem cells (CSCs) [23–26]. There is a strong connection between mitophagy and cellular adaptability. Notably, a metabolic shift induced by mitophagy from glycolysis to oxidative phosphorylation enhances the stemness characteristics of CSCs [27]. CSCs undergo specific metabolic changes governed by mitochondrial dynamics, shaped by the cellular microenvironment. It has been documented that mitochondrial function contributes to the metabolic reprogramming of CSCs in nasopharyngeal carcinoma [28]. The switch from oxidative phosphorylation to glycolysis is critical for bolstering the stem-like qualities of CSCs. Additionally, mitophagy plays a dual role in CSCs by preserving their drug resistance and supporting the maintenance of their stemness, as well as tumor proliferation. Publications indicate that mitophagy bolsters both the stem-like properties and chemotherapeutic resilience of CSCs in oral squamous cell carcinoma [29]. Furthermore, curtailing Drp1 function by inhibiting COX-2 has been demonstrated to diminish stem-like qualities, which in turn makes nasopharyngeal carcinoma cells more receptive to the effects of 5-fluorouracil [28]. Hence, within the realm of oncological treatment, pinpointing biomarkers related to mitophagy might steer towards therapies that are more precise, efficacious, and lower in toxicity.

In our study, we initially explored the expression levels and prognostic value of mitophagy-related genes at the pan-cancer level. Subsequently, we integrated multi-omics data to elucidate the molecular characteristics, biological functions, infiltration levels in the tumor microenvironment, and clinical significance of different types of mitophagy alterations in HNSCC. Furthermore, we developed and validated a prognostic tool named Mitophagy Subgroup Risk Score (MSRS), aimed at predicting the prognosis and immune response of HNSCC patients, and applied it at the single-cell level to explore intercellular communication. Concurrently, laboratory experiments validated the biological function of SLC26A9, one of the genes in MSRS.

## MATERIALS AND METHODS

### Collection and management of data

The gene expression profiles and clinical information of HNSCC were downloaded from the UCSC Xena database [30]. Copy number variation data for head and neck squamous cell carcinoma were downloaded from the TCGA website using the R-package TCGAbiolinks. Tumor immune cycle scores were downloaded from the TIMER2.0 database. The specific transcription factors for HNSCC (Supplementary Table 1) were downloaded from the HumanTFDB (http://bioinfo.life.hust.edu.cn/HumanTFDB) database.

### Categorization of mitophagy subtypes in HNSCC

A total of 29 Mitophagy-Related Genes (MRGs) (Supplementary Table 2) were retrieved from databases such as Reactome, CPDB, KEGG, and MSigDB [31–34]. Using R package ‘corrplot’, we evaluated correlations among these genes. HNSCC patients were classified into mitophagy subgroups (k=3) using the “ConsensusClusterPlus” R package [35]. Subsequently, differential expression genes between subtypes were analyzed using the “limma” R package.

### Pathway enrichment in subgroups

Pathway enrichment was performed using the GSVA method through the GSVA R package, with pathway enrichment data sourced from gmt files in the MSigDB [31] and ConsensusPathDB [33] databases.

### Analysis of the tumor microenvironment (TME)

We assessed the immune cell composition in the TME of various subgroups using algorithms such as TIMER, CIBERSORT, QUANTISEQ, MCPCOUNTER, XCELL, and EPIC. Additionally, ssGSVA was applied to validate differences in immune infiltration across HNSCC subgroups [36–39]. The infiltration levels based on immune and stromal scores in HNSCC were calculated using the “ESTIMATE” R package.

### Mutation profile analysis in subgroups

We used the “Maftools” R package to compare mutation patterns across different subgroups [40]. Functions within “Maftools” were used to calculate mutation spectra for different mitophagy subgroups and to explore drug-gene interactions and carcinogenic pathways [41]. Additionally, somatic copy number alterations were analyzed using GISTIC 2.0 [42].

### Evaluation of chemotherapy response disparities among subgroups

Based on the GDSC database [43], we used the “oncoPredict” R package [44] to assess differences in drug sensitivity among subgroups. Furthermore, the CellMiner [45] and CCLE [46] databases were used to analyze the correlation between mitophagy-related genes and drug sensitivity in HNSCC cell lines.

### Machine learning algorithms

To construct the Mitophagy Subgroup Risk Score (MSRS), we integrated ten different machine learning algorithms, including RSF, Enet, Lasso, Stepwise Cox, Ridge, CoxBoost, plsRcox, SuperPC, GBM, and survival SVM. We applied 94 different combinations of these algorithms to identify the one with the highest average concordance index (C-index) across multiple cohorts. The “timeROC” R package was used to calculate the Area Under the Curve (AUC) to verify the accuracy of the MSRS. Additionally, the independent predictive capability of the MSRS was confirmed using Cox regression analysis in the “survival” R package.

### Cell–cell communication analysis

We used GSE181919 dataset to explore the role of mitophagy genes at the single-cell level. R package “Seurat” was used to perform dimension reduction and clustering analysis, and the annotation of cell cluster was obtained by R package “SingleR”. We employed the ‘CellPhoneDB’ package [47] to explore communication at the cellular level between immune and HNSC cells, concentrating on the identification of distinct ligand-receptor pairs.

### Immunotherapy efficacy prediction

We used the Tumor Immune Dysfunction and Exclusion (TIDE) algorithms [48, 49] to predict responses to immune checkpoint blockade (ICB) therapy. The patient cohorts for this immunotherapy study were derived from the TIGER database [50], which includes the GSE91061 (melanoma) and phs000452 (melanoma) datasets.

### Cultivation of cell lines

HNSC cell lines including SCC15 and HN30 were obtained from the public laboratory of Tianjin Medical University Cancer Institute and Hospital. For their cultivation, we used DMEM enriched with 10% FBS and 1% penicillin-streptomycin. The incubation conditions for these cell lines were carefully controlled, while the environment was maintained at constant temperature of 37° C with an atmosphere of 5% CO_2_, ensuring optimal growth conditions and allowing for accurate and reliable experimental results.

### siRNA transfection

For transfection studies, SCC15 and HN30 cell lines were seeded into 6-well plates. According to the protocol provided by the supplier, transfection was carried out using si-SLC26A9-1 (sequence 5’- CAGCCAAGAUCAAAGCUGUGGUGUU -3’), si-SLC26A9-2 (sequence 5’- GGGCUUCAUGCAGUUUGGCUUUGUG -3’), and a non-targeting siRNA control (sequence 5’- CAGAAAGCUAAGAUCUGGGUCCGUU-3’) with Hieff Trans® Liposomal Transfection Reagent (Yeasen, China). The knockdown efficiency was verified by Western blot 48 hours post-transfection.

### Cell viability assay

Cells were collected through a process of digestion followed by centrifugation. After counting the cells, they were seeded into 96-well plates at a density of 2,500 cells per well. To assess cell viability, we utilized a Cell Counting Kit-8 (ApexBio, USA), conducting measurements at intervals of 0, 24, 48, and 72 hours according to the instructions provided with the kit. This methodical approach allowed us to accurately monitor the health and proliferation of the cells over time.

### Cell lysis and western blotting

Cell lysis for protein extraction was performed at cold temperatures using a lysis buffer containing phosphatase and protease inhibitors. Protein levels were quantified with the SDS. The proteins were separated by 4-12% SDS-PAGE, transferred to PVDF membranes, blocked, and incubated with primary and secondary antibodies sequentially. Detection of the target proteins was achieved using a chemiluminescence reagent. The primary antibodies applied were anti-SLC26A9 (ABclonal, A18530) and anti-beta-actin (Santa, sc-8432).

### Transwell assays and wound healing assays

SCC15 and HN30 cells, post transfection with either si-SLC26A9-1, si-SLC26A9-2, or si-NC, were harvested, washed with PBS, and subsequently resuspended in DMEM medium. These cells were then placed into the upper chamber of a 24-well plate which contains an insert with 8 μm pore size. In the lower chamber, 700 μL of DMEM containing 10% FBS was added. Following a period of 24 hours, cells remaining on the upper surface were wiped off, and those adhered to the lower surface were fixed with 4% paraformaldehyde (PFA) and stained with crystal violet for further image acquisition and analysis.

### Wound healing assay

SCC15 and HN30 cells transfected with siRNA were cultured in 6-well plates to near confluence. Subsequently, a sterile pipette tip was used to create scratches through the monolayer. Photographs were taken immediately (0 hours) and at 48 hours after the scratch was made. The healing progress was quantified using the ImageJ software.

### Colony formation assay

To assess the colony formation capacity of the cells, we carefully seeded between 800 to 1000 cells in each well of 6-well plates. These were then incubated at a consistent temperature of 37° C for a duration of 10 to 14 days to allow sufficient time for colony development. Following the incubation period, we fixed the colonies using methanol for 15 to 30 minutes, ensuring their preservation for analysis. This step was followed by staining with 0.1% crystal violet for 15 minutes to highlight the colonies for easier counting. The final step involved meticulously counting the colonies, providing a clear measure of the cells’ ability to proliferate and form colonies under the given conditions.

### EdU assay

After transfection process the cells were plated into 24-well plates (5 *10^4^ cells) and cultured overnight in a 37° C incubator. Following the protocol provided by the BeyoClick EdU Cell Proliferation Kit with Alexa Fluor 488, EdU detection was performed, EdU-positive cells were stained with Azide 488 and Hoechst 33342 to differentiate them from non-proliferative cells. We captured images from three randomly selected fields of view under a microscope to ensure a representative sampling. The percentage of EdU-positive cells was calculated by the following formula: EdU-positive rate = EdU-positive cell count/(EdU-positive cell count + EdU negative cell count) × 100%.

### Statistical analysis

Experimental results are presented as mean ± standard deviation. We employed the chi-squared test to explore differences in categorical variables, including clinical characteristics, among various subgroups to identify significant variations P-value of less than 0.05 was considered to indicate statistical significance. The Benjamini-Hochberg (BH) method was applied to calculate adjusted P-values for multiple testing corrections. All data processing, statistical analyses, and the generation of graphical representations were conducted using R software (version 4.1.3), ensuring rigorous and comprehensive analysis.

### Availability of data and materials

The open-access datasets are available through the following URL: GSE41613, GSE42743, GSE65858, and GSE181919 (https://www.ncbi.nlm.nih.gov/geo/) and the Cancer Genome Atlas (TCGA) HNSC project (http://xena.ucsc.edu/).

## RESULTS

### The role of mitophagy in cancers and its impact on patient survival

**Figure 1:**
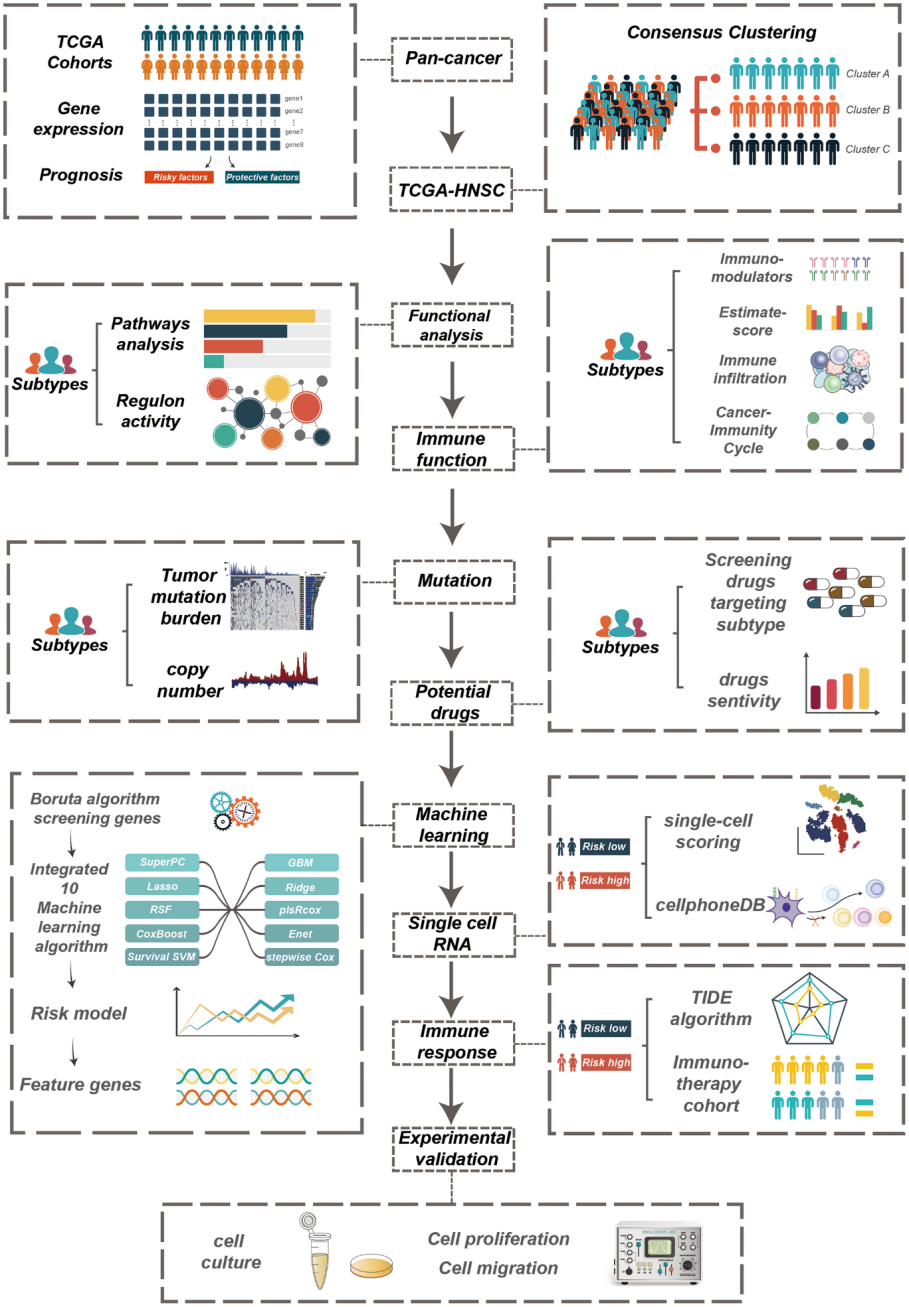
The workflow of the study.

Figure 1 illustrates the sequential steps of our research process. We investigated the complex regulation of mitophagy in various cancer types by assessing the expression levels of mitophagy-related mRNAs in diverse cancer types. Our findings indicate that the expression levels of CSNK2B, MTERF3, and PGAM5 are elevated in multiple cancers, while the expression levels of PINK1 and PRKN are reduced in several cancers (Supplementary Figure 1A). We used Spearman (upper right) and Pearson (lower left) correlation analysis to study the relationship between Mitophagy-Related Genes (MRGs) in the TCGA-HNSCC dataset, identifying significant associations between RPS27A and TOMM22, TOMM7, UBA52 (Supplementary Figure 1B). Furthermore, our study revealed the connection between gene expression profiles and patient prognosis (Supplementary Figure 1C), finding that most MRGs act as risk factors in HNSC, LIHC, KICH, LUAD, LUSC, BRCA, while TOMM6 serves as a protective factor in THYM, OV, DLBC, and PINK1 as a protective factor in KIRC, KIRP, ACC.

### Copy number and mutation events of mitophagy regulators in cancers

**Figure 2:**
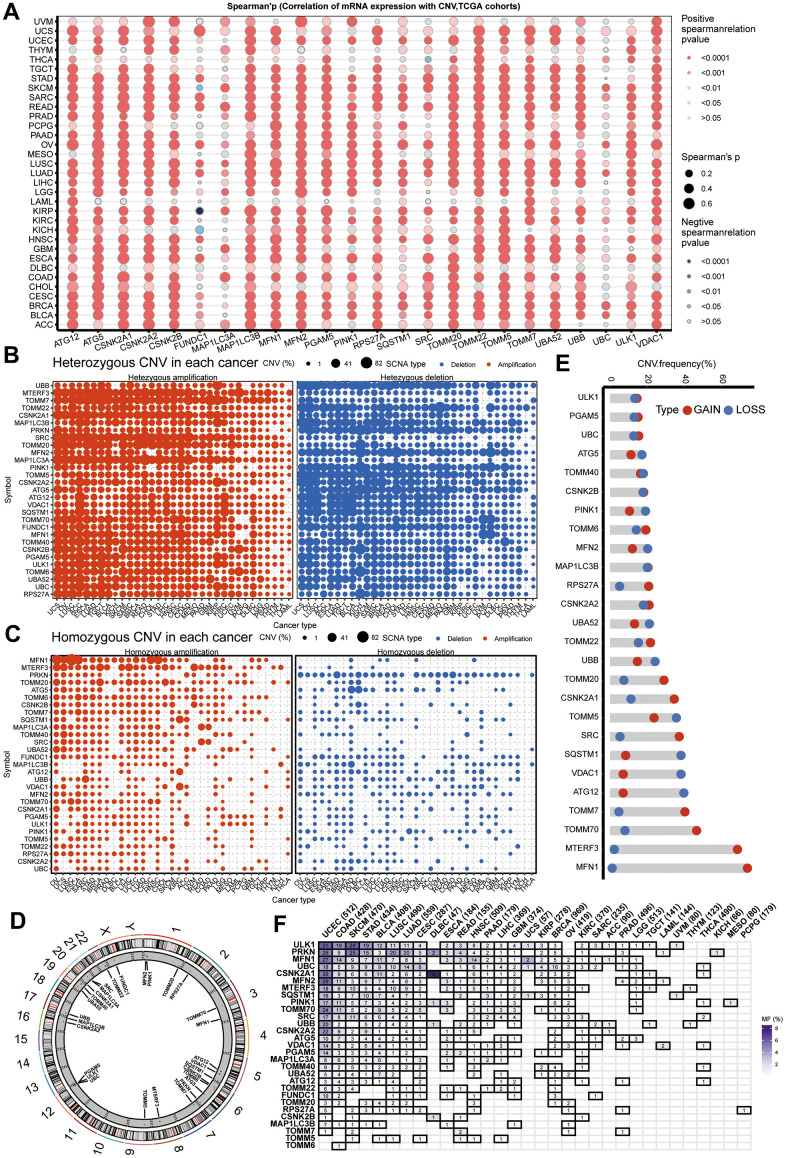
**CNV and sequence alteration contribute to abnormal mitophagy genes Levels.** (**A**) CNV strongly correlates to gene expression of mitophagy regulators in pan-cancer using spearman analysis. (**B**, **C**) Heterozygous and homozygous amplification/deletion of mitophagy regulators in pan-cancer. Amplification, red; Deletion, blue. (**D**) The location of CNV of mitophagy regulators on 23 chromosomes. (**E**) CNV of mitophagy regulators in TCGA-HNSC dataset. CNV loss, blue; CNV gain, red. (**F**) Mutation frequency of mitophagy regulators in pan-cancer.

To further explore the reasons for changes in mitophagy-related genes, we validated copy number variations (CNV) in cancer and observed a significant positive correlation between CNV and mRNA expression (Figure 2A). As shown in Figure 2B, genes such as MTERF3, TOMM7, CSNK2A1, SRC, TOMM20, and MAP1LC3A commonly exhibit heterozygous amplification in tumors, whereas UBB, TOMM22, MAP1LC3B, and PRKN mainly show heterozygous deletion. However, homozygous amplifications and deletions were found to be infrequent (Figure 2C). Figure 2D displays the genomic locations of CNVs in mitophagy-related genes. Notably, in HNSCC, genes such as TOMM7, TOMM70, MTERF3, and MFN1 have a higher frequency of CNV amplification, while SQSTM1, VDAC1, and ATG12 have a higher frequency of CNV deletion (Figure 2E). Furthermore, we delved into the mutation status of mitophagy-related genes and found that these genes have a higher mutation frequency in UCEC, COAD, SKCM, STAD, BLCA, LUSC, and LUAD, but mutations are rare in PCPG, MESO, and KICH (Figure 2F).

### Defining three subgroups within HNSCC based on mitophagy regulator expression

**Figure 3:**
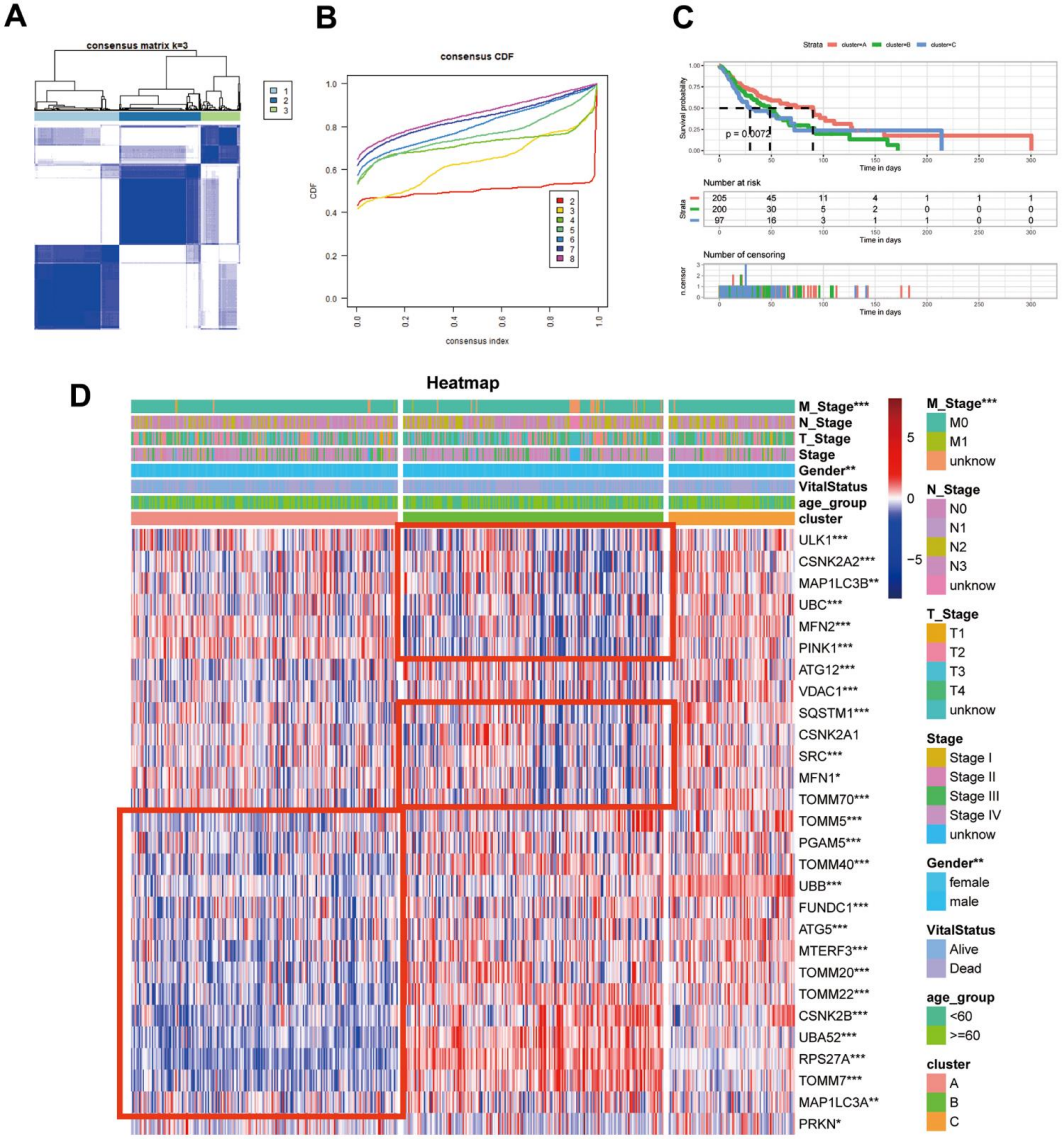
**Identification of mitophagy subtypes of HNSCC.** (**A**) Consensus matrix of samples in TCGA-HNSC for k=3. (**B**) The cumulative distribution function curves for k = 2 to 8. (**C**) Kaplan-Meier survival analysis for overall survival of the three subtypes in TCGA-HNSC dataset. (**D**) The expression profiles of the mitophagy regulators in three subtypes and normal kidney samples. Statistical significance denoted as ****p < 0.0001, ***p < 0.001, **p < 0.01, *p < 0.05.

We employed an unsupervised clustering algorithm to divide TCGA-HNSCC samples into three distinct molecular subgroups based on the expression profiles of mitophagy-related genes (Figure 3A, 3B). Patients in Cluster A exhibited a significantly enhanced survival advantage compared to those in the other two clusters (Figure 3C). Additionally, within Clusters B and C, a specific subset of mitophagy related genes shown not significantly higher expression levels. TOMM20, TOMM22, CSNK2B, UBA52, RPS27A, TOMM7 were less expressed in Cluster A, while in Cluster B, the expression levels of ULK1, CSNK2A2, MAP1LC3B, UBC, MFN2, PINK1 were lower. However, the expression of mitophagy-related genes was higher in Cluster C (Figure 3D).

### Enrichment analysis of functional pathways in varied patterns of mitophagy alterations

We employed Gene Set Variation Analysis (GSVA) to assess metabolic pathway signatures. Cluster A was characterized by metabolic dormancy, showcasing downregulation in pathways such as glucose, amino acid, nucleotide, and RNA metabolisms, the TCA cycle, and tyrosine metabolism. Conversely, Clusters B and C demonstrated metabolic exuberance with these signatures predominantly active, hinting at an augmented metabolic state (Supplementary Figure 2A). Furthermore, GSVA revealed a consistent enrichment of the hypoxia signature in Cluster B (Supplementary Figure 2B), a condition known to hinder cancer immunotherapy effectiveness due to reduced oxygen levels in tumors [51–53]. Addressing hypoxia might thus potentiate immunotherapeutic outcomes for patients in Cluster B. Additionally, an attenuated exosomal signature in Cluster B implicates a potential interplay between mitophagy and exosome dynamics (Supplementary Figure 2B).

To further our transcriptomic investigation, we harnessed RTNduals [54], an R-based analytical package, to decipher transcription factor regulons specific to mitophagy subtypes, sourced from the HumanTFDB. Notably, ZFP36L1 activity was minimal in Cluster B, suggesting a dampened cell cycle mechanism in this subgroup (Supplementary Figure 2C). Given recent insights linking ZFP36L1 [55] to immune infiltration in tumor microenvironments, these findings suggest that alterations in mitophagy may regulate crucial biological functions.

### Assessing distinct immune profiles across mitophagy subgroups

**Figure 4:**
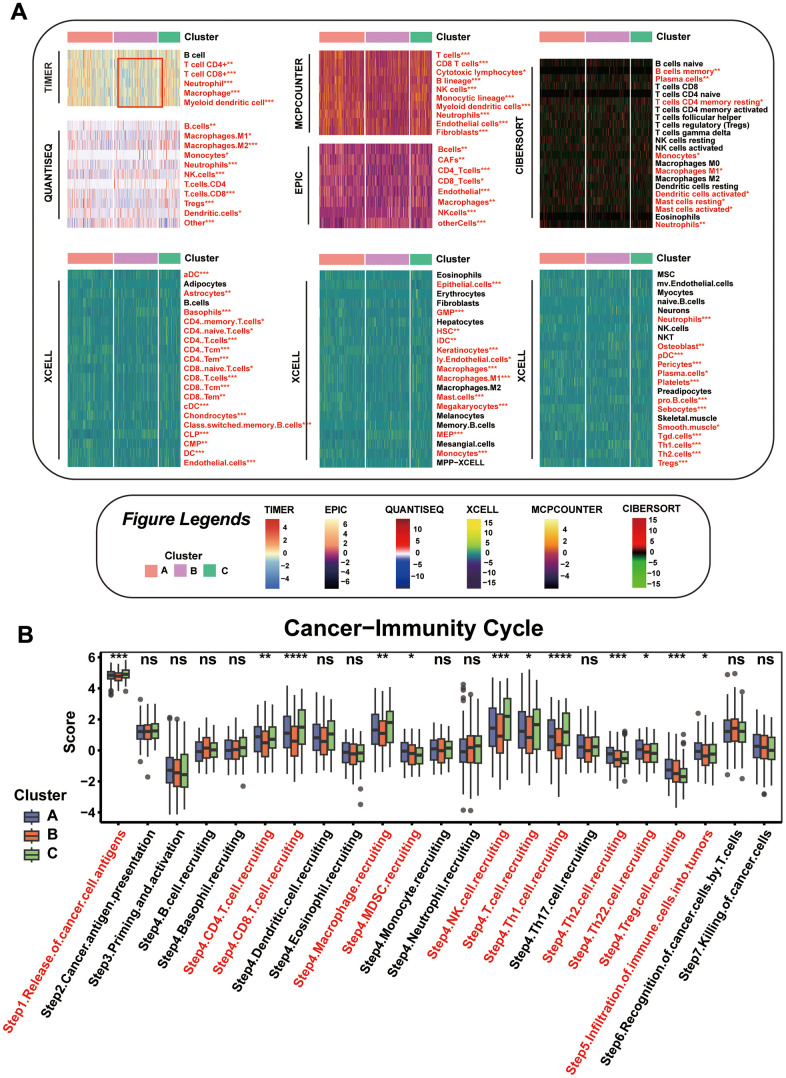
**Comparison of the Specific Immune Infiltration Landscape among Three Subgroups.** (**A**) Heatmap of immune cell infiltration among the subtypes. (**B**) Boxplot of cancer immunity cycle in three mitophagy modification subtypes. Statistical significance denoted as ****p < 0.0001, ***p < 0.001, **p < 0.01, *p < 0.05.

To compare immune activity among various subgroups, we evaluated immune process enrichment scores across different subgroups using Gene Set Variation Analysis (GSVA). Notably, Cluster B was marked by a pronounced decrease in pathways related to chemokines, chemokine receptors, immune inhibitors, and immune stimulators (Supplementary Figure 3A). Further analysis of the tumor microenvironment (TME) cell composition revealed a substantial reduction in immune cell infiltration in Cluster B compared to Clusters A and C, as shown in Figure 4A. This suggests that Cluster B might represent an ‘immune desert’ phenotype with notably reduced immune engagement.

This observation is in line with survival analyses, where Cluster B patients had poorer outcomes compared to those in Cluster A. Our focus then turned to dissecting the anti-cancer immune cycle, which involves multiple critical steps. In Cluster B, there was a notable decrease in key phases of this cycle, including Cancer cell antigens are released (Step 1), and immune cells such as CD4 and CD8 T cells, macrophages, NK cells, Th1, Th2, and Th22 cells are mobilized (Step 4), along with their infiltration into the tumor (Step 5), as depicted in Figure 4B.

### Tumor genomic alterations and CNV profiles in three mitophagy subgroups

Among the three defined groups, we analyzed the distribution of tumor somatic mutations. Supplementary Figure 4A–4C show the top 25 most frequently mutated genes, indicating a high mutation burden across all subgroups. Additionally, we assessed druggable targets based on these mutations, using the DGIdb and the maftools package to investigate drug-gene interactions. Potential therapeutic targets related to the three mitophagy modification patterns were categorized into 19, 18, and 20 groups, which included targets within clinically relevant, druggable genome, kinase, and histone modification categories, as detailed in Supplementary Figure 4D–4F. Employing the R package maftools, we examined less common genetic changes in cancer pathways including RTK-RAS, Hippo, WNT, TP53, and the Cell Cycle. In Cluster A, the NRF2, TP53, and TGF-Beta pathways were most affected, while Cluster B showed a pronounced impact on the NRF2 and TGF-Beta pathways. Cluster C affected the NRF2 and RTK-RAS pathways most significantly, as shown in Supplementary Figure 4G–4I.

**Figure 5:**
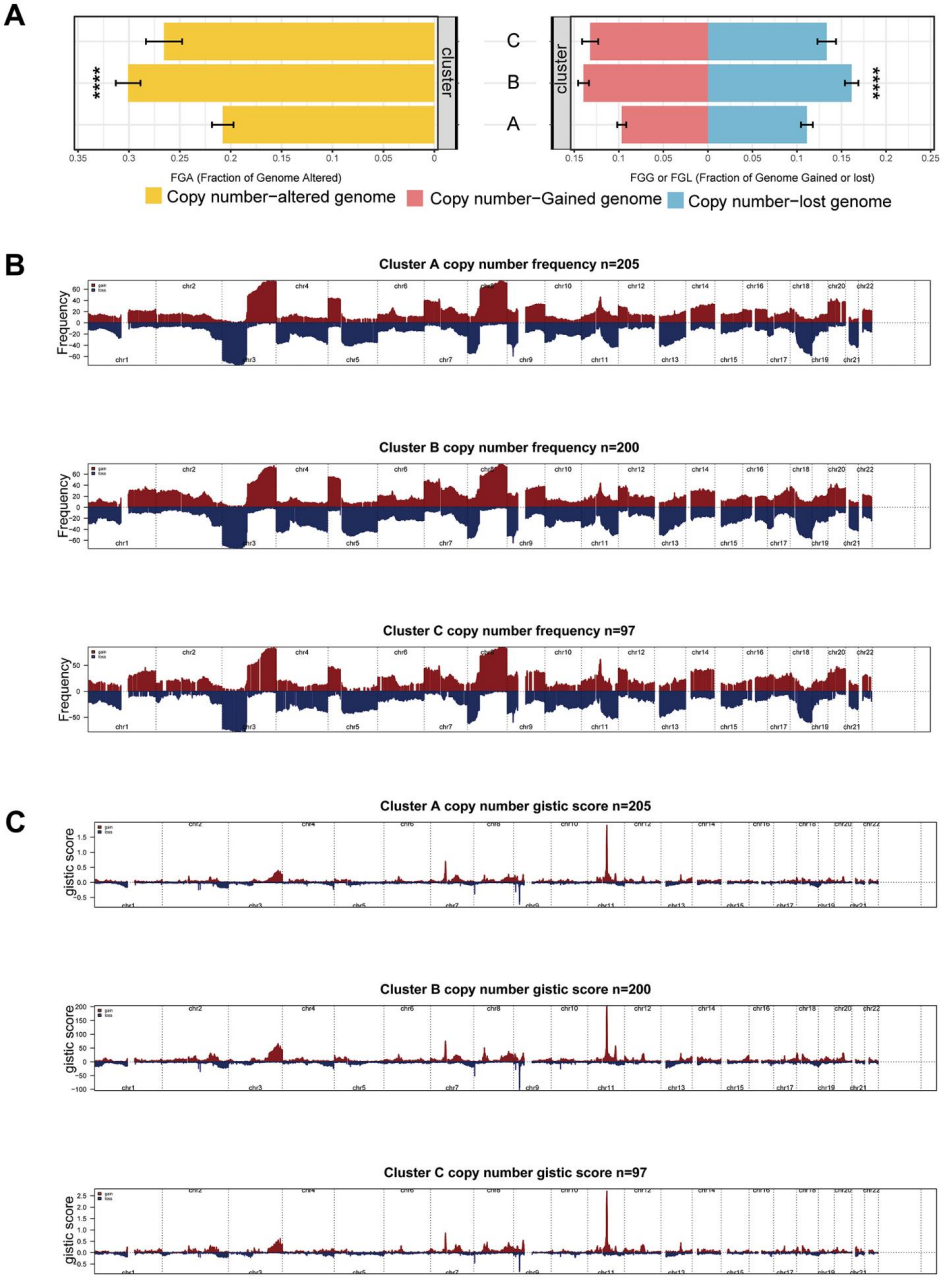
**CNV differences among subgroups.** (**A**) CNV rate among subgroups. (**B**, **C**) Differences of gain/loss percentage (**B**) and GISTIC score (**C**) in Cluster A, B, and C. Statistical significance denoted as ****p < 0.0001, ***p < 0.001, **p < 0.01, *p < 0.05.

In our comparative assessment of Copy Number Variation (CNV) among the clusters, Cluster B showed the highest rate of CNV, with Clusters C and A following closely, as demonstrated in Figure 5A. This observation is supported by similar trends in the percentage of gain/loss and GISTIC scores for amplification and deletion regions on chromosomes analyzed using GISTIC 2.0, as depicted in Figure 5B, 5C. These insights suggest that diverse CNVs play a role in defining the distinct mitophagy-related tumor subtypes.

### Chemotherapeutic response variations among mitophagy subgroups

We conducted a comparative analysis of drug sensitivity using IC_50_ values obtained from the GDSC database. Our analysis predicted enhanced sensitivity to palbociclib for patients categorized within Cluster B. It was found that gefitinib, navitoclax, and dasatinib were more potent for patients in Cluster A, while those in Cluster C were more responsive to temozolomide, cisplatin, and tamoxifen, as indicated in Supplementary Figure 5.

Subsequently, we sought to identify treatments that counteract cancer-driving processes. We examined the relationship between mitophagy gene expression and drug sensitivity using data from the CellMiner database. We found that the expression of PINK1 inversely correlates with the IC_50_ values for AFP464, palbociclib, and denileukin diftitox (Supplementary Figure 6), implying that patients with higher PINK1 expression may benefit more from these drugs. Additionally, Econazole nitrate and Crizotinib appeared to be more effective in individuals with lower expression of RPS27A and TOMM7, respectively.

### Construction of the mitophagy subgroup risk score (MSRS) by integrated machine learning

**Figure 6:**
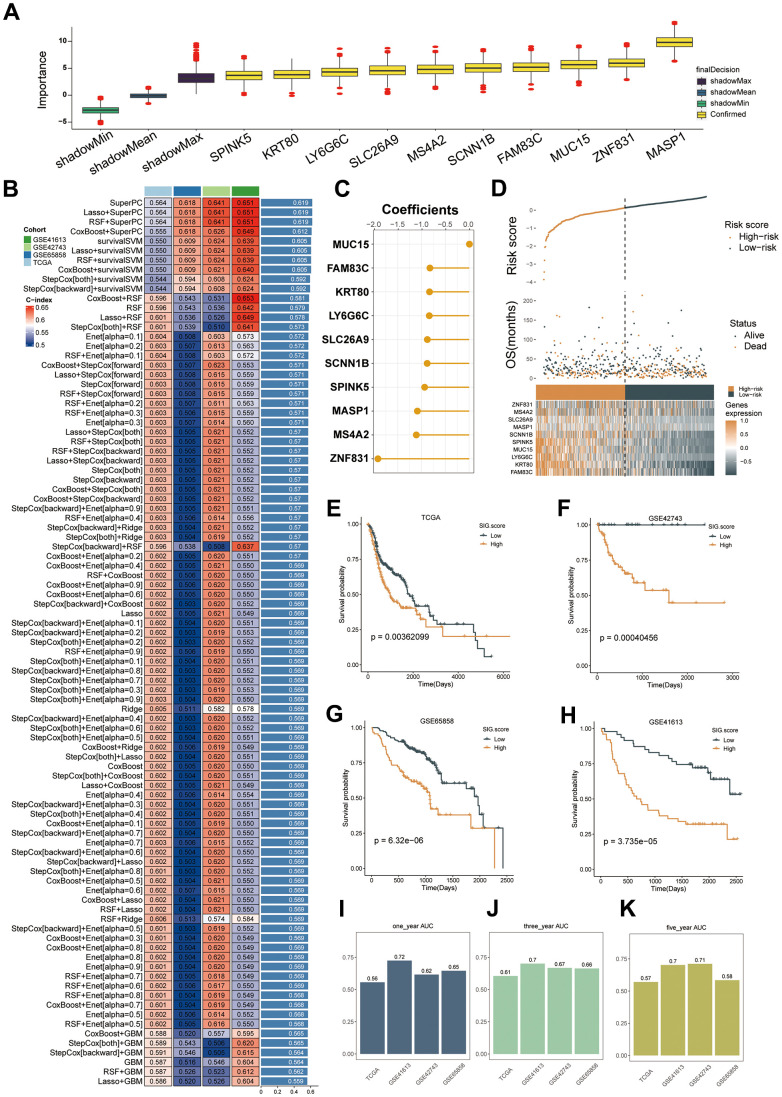
**Machine learning-based gene signatures based on mitophagy subgroups.** (**A**) The Boruta algorithm identified 10 mitophagy-related genes. Yellow represents confirmed features while other colors denote shadow attributes. The corresponding boxplots compared the concordance index (C-index) values. (**B**) Machine learning was used to build 93 different models, and their C-indices were tested in each verification set. (**C**) The coefficients of ten genes calculated by SuperPC. (**D**) Statistical analysis of the risk scores and survival status of ten genes as well as their expression characteristics in TCGA. (**E**–**H**) Prognoses of patients in the TCGA (**E**), GSE42743 (**F**), GSE65858 (**G**), and GSE41613 (**H**) sets. (**I**–**K**) Predicting patient survival at 1, 3, and 5 years using the MSRS.

By employing the Limma algorithm, the computation of unique genes for the three subcategories and the assessment of overlaps were conducted, leading to the discovery of a grand total of 468 genes linked to mitophagy subtypes (Supplementary Table 3). Univariate Cox regression analysis (Supplementary Table 4) revealed 194 genes that exhibited a significant association with overall survival. Out of the 194 prognostic genes, the bootstrap technique identified 19 genes that remained stable even after resampling the samples and were also present in the validation datasets (Supplementary Table 5). Furthermore, we employed the Boruta algorithm and narrowed down the selected genes to a group of 10 genes that were confirmed to have greater importance in terms of recurrence (Supplementary Figure 7), as illustrated. Based on their inferred degree of importance, the Boruta algorithm ranked 10 genes, identified as Figure 6A.

We employed our machine learning algorithm to scrutinize these 10 genes and develop a predictive model. Utilizing 94 forecasting models, we examined the TCGA dataset and calculated the concordance index (C-index) for three validation datasets. Ultimately, the integration of the SuperPC algorithms yielded the most effective prototype, achieving an average C-index of 0.619 across all validation datasets, as illustrated in Figure 6B. The SuperPC algorithm revealed 10 crucial genes for determining the risk score, as depicted in Figure 6C. The calculation of MSRS involved the evaluation of the expression levels of these 10 genes and their respective regression coefficients for every individual within the mitophagy subgroup.

As Figure 6D–6H, there was a correlation between an escalation in the MSRS, a decrease in the overall survival (OS), and an elevation in the mortality rates. Patients were categorized into high-risk and low-risk groups based on the median MSRS value. Consistently, the TCGA training dataset and the three validation datasets showed that the high-risk group had significantly worse survival outcomes than the low-risk group, as depicted in Figure 6E–6H. In TCGA-HNSC, the ROC analysis verified that the MSRS has a robust ability to discriminate, as evidenced by AUC values of 0.56, 0.61, and 0.57 for one-, three-, and five-year survival, respectively. displays the AUCs for GSE41613 as 0.72, 0.7, and 0.7; for GSE42743 as 0.62, 0.67, and 0.71; and for GSE65858 as 0.65, 0.66, and 0.58.

### Interactions between immune cells and HNSC cells that involve ligand-receptor pairs

To investigate the potential interaction between immune cells (T cells, B cells, etc.) and head and neck squamous cell carcinoma (HNSC) cells, we utilized single-cell RNA sequencing (scRNA-seq) to analyze their collaboration. Initially, we calculated risk scores for various cell types and discovered that epithelial cells exhibited the lowest risk score, as illustrated in Supplementary Figure 8A. Supplementary Figure 8B shows that risk scores were higher in tumor tissue-associated cells than in normal tissues.

Afterwards, we conducted a thorough analysis of the associations between high-risk epithelial cells (Riskhighepi) and low-risk epithelial cells (Risklowepi) along with other cellular components using CellphoneDB in combination with scRNA-seq data. As shown in Supplementary Figure 8C, when acting as a receptor or ligand, Riskhighepi has a significantly greater number of ligand-receptor pairs in interactions with other cells than Risklowepi. Supplementary Figure 8D illustrates the interaction intensity between Riskhighepi and other cell types, showing notably stronger interactions with endothelial cells, fibroblasts, and smooth muscle cells. Upon contrasting the communicative signals with Riskhighepi, we identified that the signaling pathway showing a robust interaction between Risklowepi and Smooth muscle cells involved COL17A1_a2b1_complex, COL17A1_a10b1_complex, and COL17A1_a1b1_complex (Supplementary Figure 8E). The pathway connecting Risklowepi and Fibroblasts included COL17A1_a1b1_complex, COL17A1_a11b1_complex, and MIF_TNFRSF14 (Supplementary Figure 8F), and the interaction between Risklowepi and endothelial cells featured MIF_TNFRSF10D, MIF_TNFRSF14, and COL17A1_a1b1_complex (Supplementary Figure 8G). Furthermore, we noticed that the signal indicating the most prominent connection between Risklowepi and other immune cells was linked to either COL17A1 or MIF (Supplementary Table 6).

### MSRS’s ability to predict ICB efficacy

To validate the immunotherapy predictive performance of the Mitophagy Subgroup Risk Score (MSRS), we assessed it using the TIDE website. We found that in the group with lower MSRS, the TIDE scores and exclusion scores were lower, while the dysfunction scores were higher (Supplementary Figure 9A–9C). Furthermore, individuals with decreased MSRS exhibited a decline in the infiltration of cancer-associated fibroblasts (CAFs), as illustrated in Supplementary Figure 9D–9G. In summary, these findings suggest that people with decreased MSRS scores exhibit increased responsiveness to immunotherapy.

In the GSE91061 cohort, a higher proportion of patients with lower MSRS had a response to immunotherapy (Supplementary Figure 9H). The findings from the GSE91061 and phs000452 immunotherapy cohorts also supported the idea that patients who had a low MSRS and received immunotherapy had a better prognosis than those with a high MSRS, as shown in Supplementary Figure 9I, 9J. The potential of the MSRS as a predictive indicator for the effectiveness of immunotherapy in these specific patient cohorts is emphasized by this observation.

### SLC26A9 is associated with tumor suppression in HNSC

**Figure 7:**
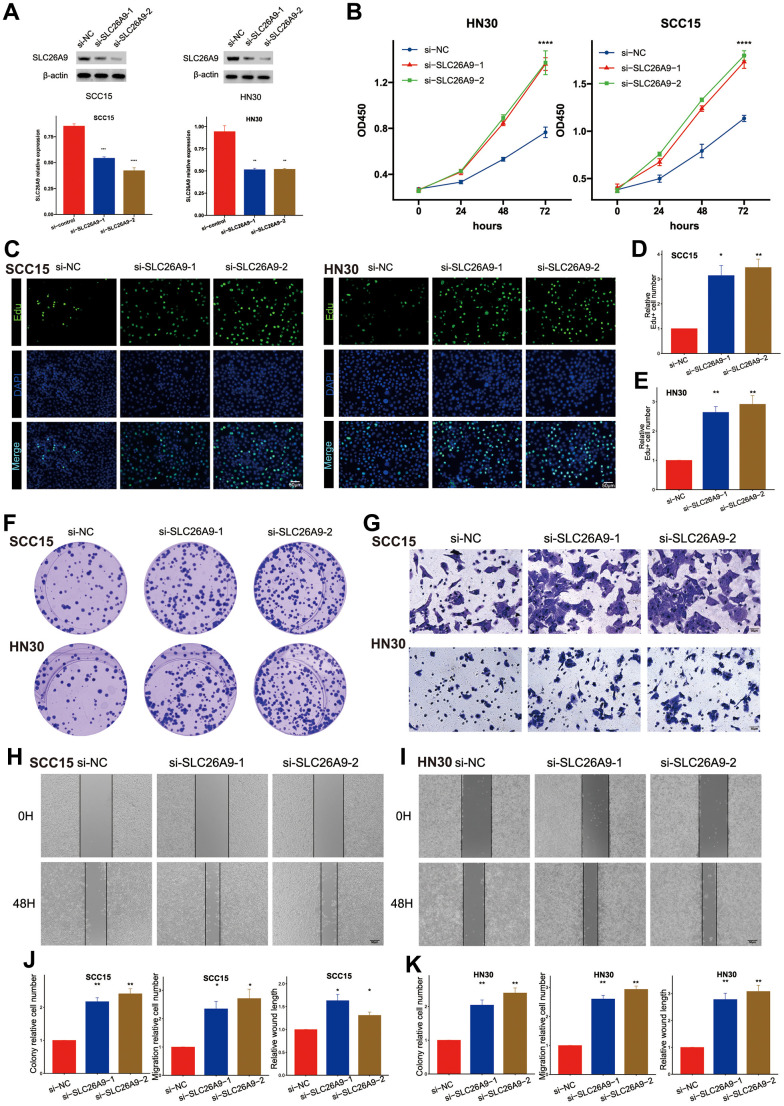
**Knockdown of SLC26A9 promoted the proliferation and migration of HNSC cells.** (**A**) SLC26A9 siRNA transfection levels analyzed via Western Blot. (**B**) CCK8 assay assessing cell viability in SCC15 and HN30 cells with reduced SLC26A9 expression. (**C**) EdU assay evaluating cell proliferation post SLC26A9 knockdown in SCC15 and HN30 cells. (**D**, **E**) Quantitative analysis of EdU-positive cell rates in SCC15 (**D**) and HN30 (**E**) cells. (**F**) Notable reduction in clone numbers in SCC15 and HN30 cells following SLC26A9 knockdown. (**G**) Transwell assay measuring migration capability of SCC15 and HN30 cells with decreased SLC26A9 expression. (**H**, **I**) Cell scratch assay examining proliferation in SCC15 (**H**) and HN30 (**I**) cells post SLC26A9 reduction. (**J**, **K**) Quantitative summary of clone numbers, migration rates in transwell assay, and wound healing rates in SCC15 (**J**) and HN30 (**K**) cells.

Through laboratory experiments, we explored the biological function of SLC26A9, which is one of the genes in the Mitophagy Subgroup Risk Score (MSRS). Supplementary Figure 10 demonstrates that SLC26A9 is a protective factor in the prognosis of HNSCC. During our research, we utilized a collection of siRNAs to inhibit the expression of SLC26A9 in SCC15 and HN30 cell lines. After 48 hours of transfection, western blot analysis confirmed the successful knockdown of SLC26A9 (Figure 7A).

Afterwards, Figure 7B shows that the decrease in SLC26A9 resulted in improved cell viability. The 5-ethynyl-2’-deoxyuridine (EdU) incorporation assay revealed enhanced cell growth in both SCC15 and HN30 cell lines upon the observation of SLC26A9 inhibition. Moreover, a rise in the quantity of colonies was observed in the cell lines when SLC26A9 was diminished, as depicted in Figure 7C–7E. Moreover, the migratory capabilities of SCC15 and HN30 cells were notably enhanced following SLC26A9 knockdown, as evidenced by transwell and wound healing assays (Figure 7G–7J). The overall results suggest that SLC26A9 inhibits the proliferation and migration of HNSC cells, highlighting its therapeutic potential as a target for addressing this type of cancer.

## DISCUSSION

Head and neck squamous cell carcinomas exhibit considerable heterogeneity. There is a critical demand for precise diagnostic tools and reliable prognostic indicators. Targeting mitophagy has gained prominence as a pivotal strategy in oncology, given its vital role in cellular survival and tumor growth. The progression of tumors has been associated with key players in the mitophagy pathway such as PINK1 [56], Parkin [57], BNIP3 [58], and FUNDC1 [59]. Therapeutic benefits have been observed by disrupting the activity of these proteins in cancer therapies. Additionally, a focus on mitophagy has often led to an increased susceptibility of cancer cells to pharmacological treatments. Inhibiting mitophagy has been noted to elevate chemosensitivity in tumor cells, reducing their ability to resist drug interventions, particularly through the action of PINK1 [60]. Moreover, blocking FUNDC1 has been noted to increase the responsiveness of cervical cancer cells to both cisplatin and radiation therapy. It has been documented that salinomycin can initiate mitophagy, which serves as a protective mechanism in tumor cells. A reduction in ATG5 has also been shown to promote apoptosis in malignant and cancer stem cells [61]. Additionally, the compound liensinine, a type of isoquinoline alkaloid, not only impedes mitophagy but also heightens the susceptibility of cancer cells to a range of anti-cancer drugs. Liensinine impedes the merging of autophagosomes with lysosomes, causing an accumulation of mitophagosomes, which consequently induces mitochondrial division through DNM1L and leads to cell death [62]. There is growing evidence that mitophagy is integrally involved in glycolysis [63], tumor development [64], activation of inflammasomes [65], and the preservation of stem cell characteristics [23, 66], via its regulatory interactions. While most research has concentrated on individual regulatory molecules, the collective impact of multiple mitophagy modulators on cancer attributes is not yet fully comprehended.

In our research, key mitophagy-related genes were scrutinized across various cancers, culminating in the discernment of three mitophagy-based classifications (Clusters A, B, and C) in individuals with HNSCC. We conducted an in-depth comparative analysis of these clusters, assessing them across multiple omics facets. This comprehensive approach resulted in the creation and validation of a prognostic tool, named MSRS, which effectively predicts patient outcomes in HNSCC.

The study exposed significant correlations between mitophagy-related genes across different cancers, suggesting a shared regulatory axis. Further scrutiny hinted that copy number variations and genetic aberrations could be driving the deregulation of mitophagy-related genes in oncogenesis. The research also unveiled distinctive clinical attributes within the three mitophagy patterns, highlighting how disruptions in mitophagy modulate the clinical trajectory of HNSCC sufferers. Notably, subjects in Cluster A manifested enhanced survival rates compared to those in Clusters B and C, which were linked with more advanced disease grades and stages.

Metabolic alteration is a hallmark of HNSCC, typifying its status as a malignant archetype [67, 68]. By conducting an enrichment analysis of transcriptomic variations, the current study identified strong links between metabolic pathways and distinct mitophagy subgroups. Metabolic functions appeared notably suppressed in Cluster A compared to Clusters B and C, pointing to metabolic intervention as a potential therapeutic avenue. Further scrutiny exposed distinct signaling dynamics within the cancer milieu among the clusters, with a marked suppression of the mitophagy signature in Cluster A. Intricate epigenetic regulatory mechanisms, involving complex modifications and interactions [69, 70], are known to initiate mitophagy, prompting our focus towards the epigenetic landscape as an actionable area. We also hypothesize that the group-specific functional disparities might be steered by key transcriptional regulators such as YBX1, TP53, ATF4, and NME2, meriting further investigation. These insights underscore the pivotal influence of mitophagy on diverse cell functions.

In head and neck squamous cell carcinoma (HNSCC), the tumor microenvironment (TME) is composed of a diverse mixture of neoplastic and assorted stromal cells, including endothelial cells, cancer-associated fibroblasts (CAFs), and components of the immune system. A prevalent feature of many HNSCC tumors is a marked immunosuppression within the TME [71, 72]. T lymphocytes and NK cells play vital roles in the TME, bolstering the host’s anti-tumor defenses. In contrast, T regulatory cells, MDSCs, and M2 macrophages play a counteractive role, promoting tumor growth. A higher presence of CD8 T cells and NK cells [73, 74] in the TME correlates with increased survival rates, while greater populations of MDSCs [75], neutrophils [76], and M2 macrophages [77] align with advanced HNSCC stages or adverse outcomes. Research indicates that mitochondrial autophagy in cancer significantly contributes to the infiltration of immune cells [65]. IL1B (interleukin 1 beta), pivotal components of the IL1 family, are associated with neutrophil migration, T cell differentiation and activation, NK cell engagement, and macrophage functions [78–80]. Growing research suggests that linear autophagy downregulates IL1B production through the regulation of NLRP3 [81–84]. IFNA and IFNB, are multifunctional cytokines enhancing antigen presentation, NK cell activities, and lymphocyte reactions [85]. The suppression of IFNA/IFNB synthesis by mitophagy was initially noted in ATG5-deficient cells, characterized by dysfunctional mitochondrial accumulation and elevated IFNA/IFNB generation [86]. Control of dysfunctional mitochondria associate mitophagy with other inflammatory cytokines, influencing immune cell infiltration. For instance, an increase in mtROS from autophagy deficiency leads to an overproduction of MIF (macrophage migration inhibitory factor) in human and mouse macrophages when stimulated by lipopolysaccharide. Besides impacting immune cell infiltration via pro-inflammatory cytokines, mitophagy can also directly exert effects on immune cells. Studies demonstrate a direct involvement of mitophagy in the development and differentiation of immune cells, such as T cells, NK cells, and macrophages. Additionally, the reduction of mitochondrial volume through autophagy is developmentally crucial for maintaining cell viability during the transformation of T cells from thymocytes to peripheral naïve T cells, as well as for the maturation of invariant NK T cells within the thymus [87, 88]. Significantly, the stage-specific control of BNIP3- and BNIP3L-mediated mitophagy is vital for the differentiation process of memory NK cells [89]. Additionally, IL10’s suppression of mTOR activity triggers mitophagy while simultaneously slowing down glycolysis in lipopolysaccharide -stimulated macrophages, as noted in studies [90, 91]. Inhibiting mitophagy with 3-methyladenine encourages a shift towards inflammatory (M1) macrophages phenotypes, whereas promoting mitophagy with rapamycin hinders M1 polarization, favoring anti-inflammatory (M2) macrophages differentiation instead [92]. Building on these insights, we extended our research to examine the impact of mitophagy on immune functionality in head and neck squamous cell carcinoma.

Cluster B was characterized by a notably sparse infiltration of immune cells, indicative of immunosuppression and the ‘cold tumor’ phenotype, which is commonly resistant to immunotherapy. Research indicates that such tumors circumvent immune detection and hinder the proliferation and activation of T cells [93]. In Cluster B, the immune response was compromised by impediments in the release of cancer cell antigens and the subsequent T-cell engagement. Improving the immune response, particularly T-cell activation and patient survival, might be feasible by leveraging dendritic cell-mediated antigen presentation within the tumor milieu [94]. This aspect aligns with findings that individuals in Cluster B faced poorer prognoses compared to those in Cluster A. Further, our study explored examined the link between mitophagy and CNV alterations, with Cluster B showing a higher incidence of CNV changes, both deletions and amplifications, while Cluster A exhibited the least CNV frequency. The progression and complexity of HNSCC are thought to correlate with CNV patterns [95], and we postulate that genetic diversity may increase the likelihood of unpredictable clinical outcomes.

As previously noted, mitophagy can modulate the effectiveness of cancer treatments [81, 96]. Our analysis revealed differential drug response profiles among HNSCC patients categorized into the various clusters. Hence, our cluster categorizations could guide more precise drug deployment in therapeutic protocols. Additionally, our research also delves into potential treatment avenues for HNSCC, particularly for those in Cluster B, aiming to advance the knowledge of the molecular dynamics that govern the efficacy of these therapeutic interventions.

Our study aimed to enrich our understanding of HNSCC tumor biology by integrating patterns of mitophagy. To address individual variations, we amalgamated an extensive array of 94 machine learning techniques to forecast patient survival and their potential reaction to immunotherapy. Nevertheless, this research faces certain constraints. Primarily, our conclusions were drawn using thorough bioinformatic methods. There remains a need for experimental corroboration, especially in understanding the interplay of mitophagy regulators and the downstream pathways they influence. Additionally, despite observed variances in drug sensitivity across the subgroups, experimental confirmation is crucial. Furthermore, while we have validated our prognostic model, certain unavoidable confounders, like ethnicity and geographical location, may have introduced bias. For more robust conclusions, further independent datasets would be beneficial.

In closing, our research dissected the implications of mitophagy in HNSCC, distinguishing three mitophagy-related subgroups. We conducted an all-encompassing assessment of these subgroups’ clinical profiles, biological roles, immune infiltration, genetic attributes, and drug response patterns. Moreover, we constructed a solid mitophagy-based prognostic framework for forecasting HNSCC patient outcomes. The insights from our study offer a novel perspective on the mitophagy-HNSCC nexus, potentially aiding in clinical strategy formulation.

## CONCLUSIONS

The MRGs were regarded as highly correlated with the prognosis of HNSCC and were recognized as exceptional prognosticators for HNSCC. Specific metabolic pathways and outcomes were linked to unique variations in mitophagy. Immune cell infiltration was more severe in subtypes that demonstrated increased levels of mitophagy. Moreover, there were differences in the way drugs responded among various subgroups. The Mitophagy Subgroup Risk Score (MSRS) is capable of effectively forecasting the immune treatment response and prognosis in patients with HNSCC. The Mitophagy Subgroup Risk Score (MSRS) gene SLC26A9 can inhibit HNSCC cell proliferation and migration. Examining the terrain of mitophagy alteration will increase understanding and further enrich the comprehension of HNSCC characterization. Additionally, it will provide guidance for future clinical decision-making.

## Supplementary Material

Supplementary Figures

Supplementary Tables 1 and 2

Supplementary Table 3

Supplementary Table 4

Supplementary Table 5

Supplementary Table 6
