# Supplementary material for: Multi omics analysis of mitophagy subtypes and integration of machine learning for predicting immunotherapy responses in head and neck squamous cell carcinoma
Source: Aging (Albany NY). 2024 Jun 21;16(12):10579–614. doi: 10.18632/aging.205964 (PMC11236326; doi:10.18632/aging.205964)
Supplement: Supplementary Figures [file aging-16-205964-s001.pdf]

SUPPLEMENTARY FIGURES

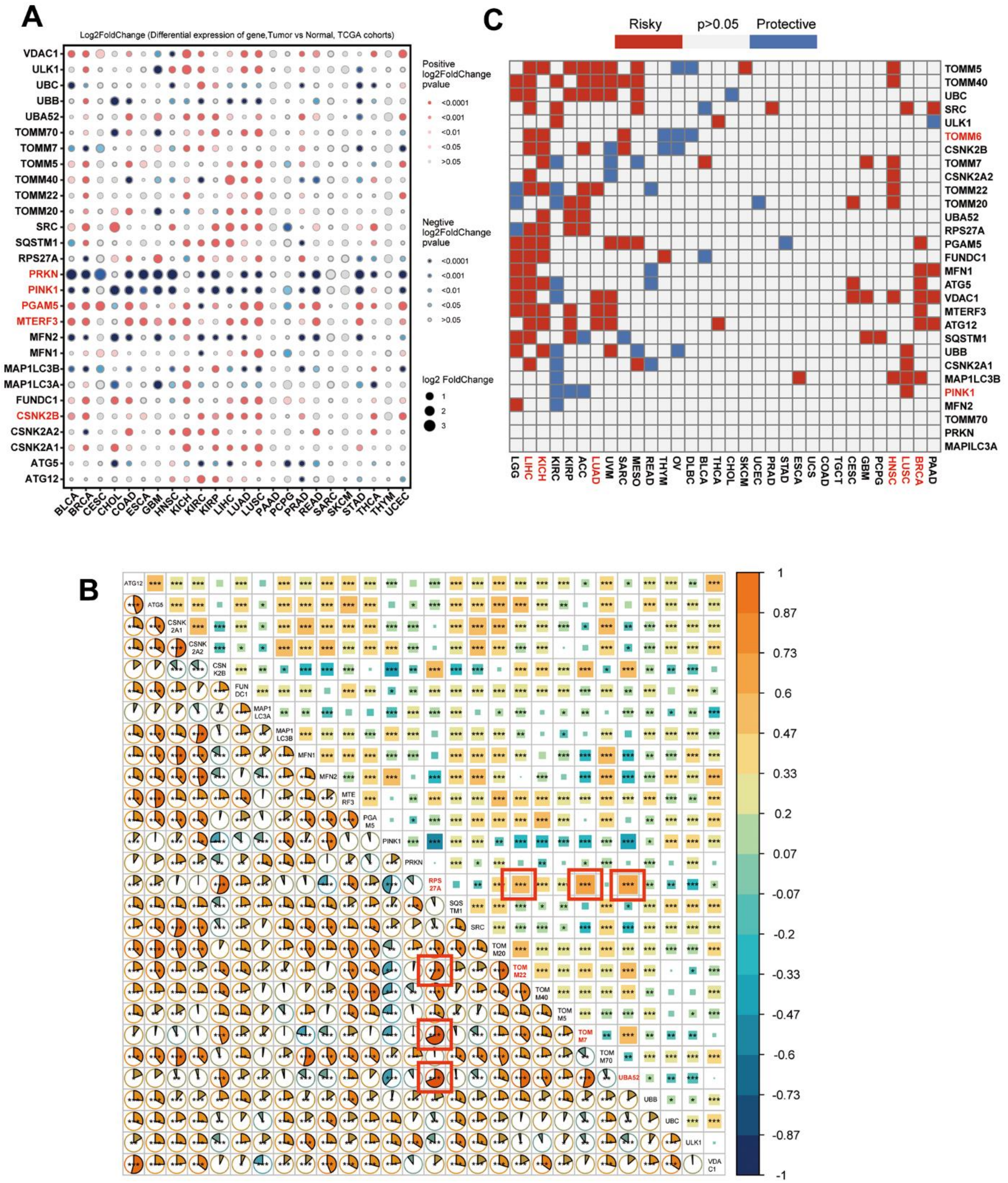

Supplementary Figure 1. Disrupted mitophagy regulators in cancers and correlations with patient outcomes: (**A**) mRNA expression related to mitophagy in multiple cancers. (**B**) The correlation of mitophagy-related genes in the expression matrix TCGA-HNSC using two methods, Spearman (upper right) and Pearson (lower left) correlation tests. (**C**) The association between transcript levels and patient outcomes.

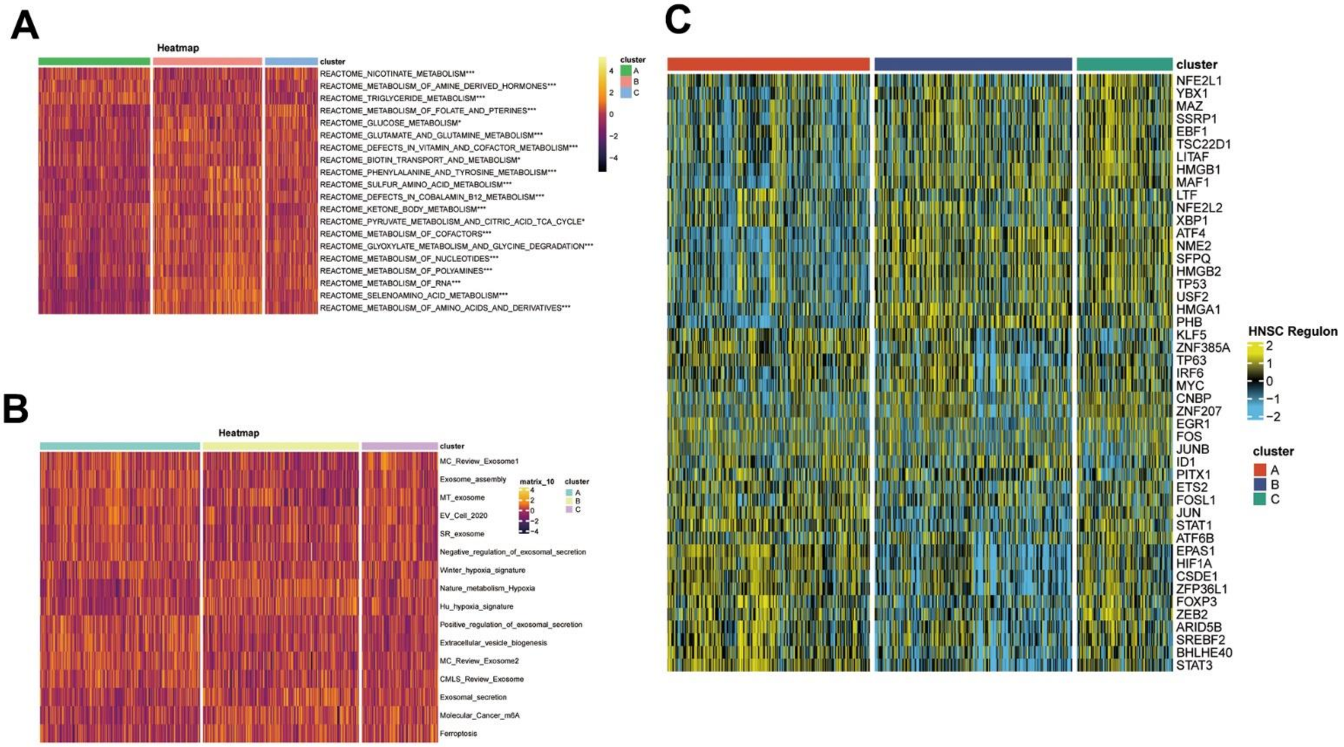

Supplementary Figure 2. Functional Enrichment Analysis in Distinct Mitophagy Modification Patterns: (**A**, **B**) Heatmap of metabolism-related and cancer-related pathway enrichment scores among the subtypes by GSVA analysis. (**C**) Heatmap of transcription factor regulon activation in three mitophagy modification subtypes. ****P < 0.0001, ***P < 0.001, **P < 0.01, *P < 0.05

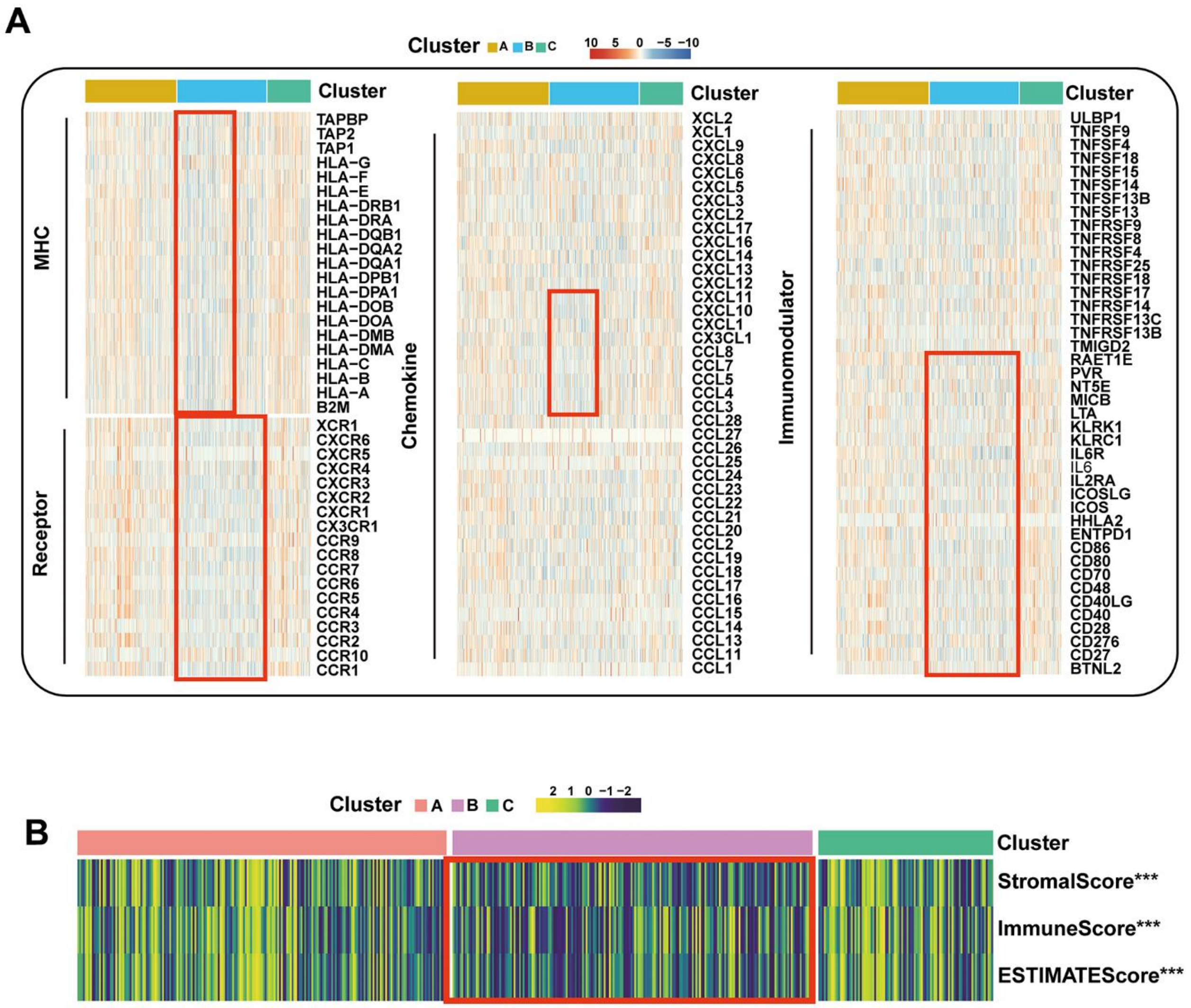

Supplementary Figure 3. Comparison of the Specific Immune Infiltration Landscape among Three Subgroups: (**A**) Heatmap of chemokines, chemokine receptors, immunoinhibitors, and immunostimulators among the subtypes by expression analysis. (**B**) Heatmap of estimate score in three mitophagy modification subtypes. ****P < 0.0001, ***P < 0.001, **P < 0.01, *P < 0.05.

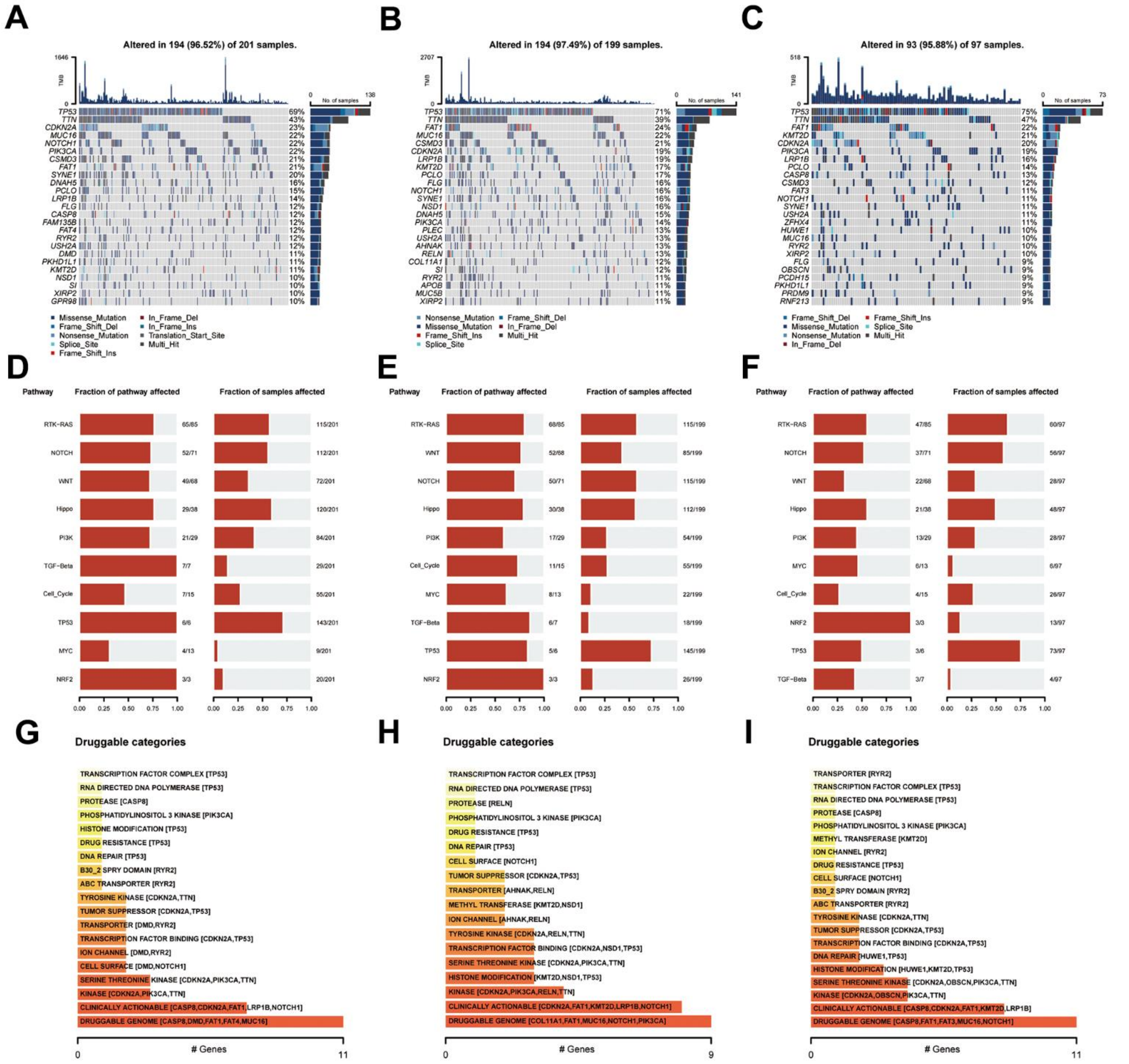

Supplementary Figure 4. Landscapes of somatic mutation among subgroups: (**A**–**C**) Waterfall plot showing the mutation patterns of the top 20 most frequently mutated genes. Each column represented patients. The upper barplot showed tumor mutational burden. The mutation frequency of each gene was indicated on the right. (**D**–**F**) Potentially druggable gene categories from mutation datasets in Cluster A, B, and C. (**G**–**I**) Onco-pathway alteration frequency and the fraction of sample affected for each pathway in Cluster A, B, and C.

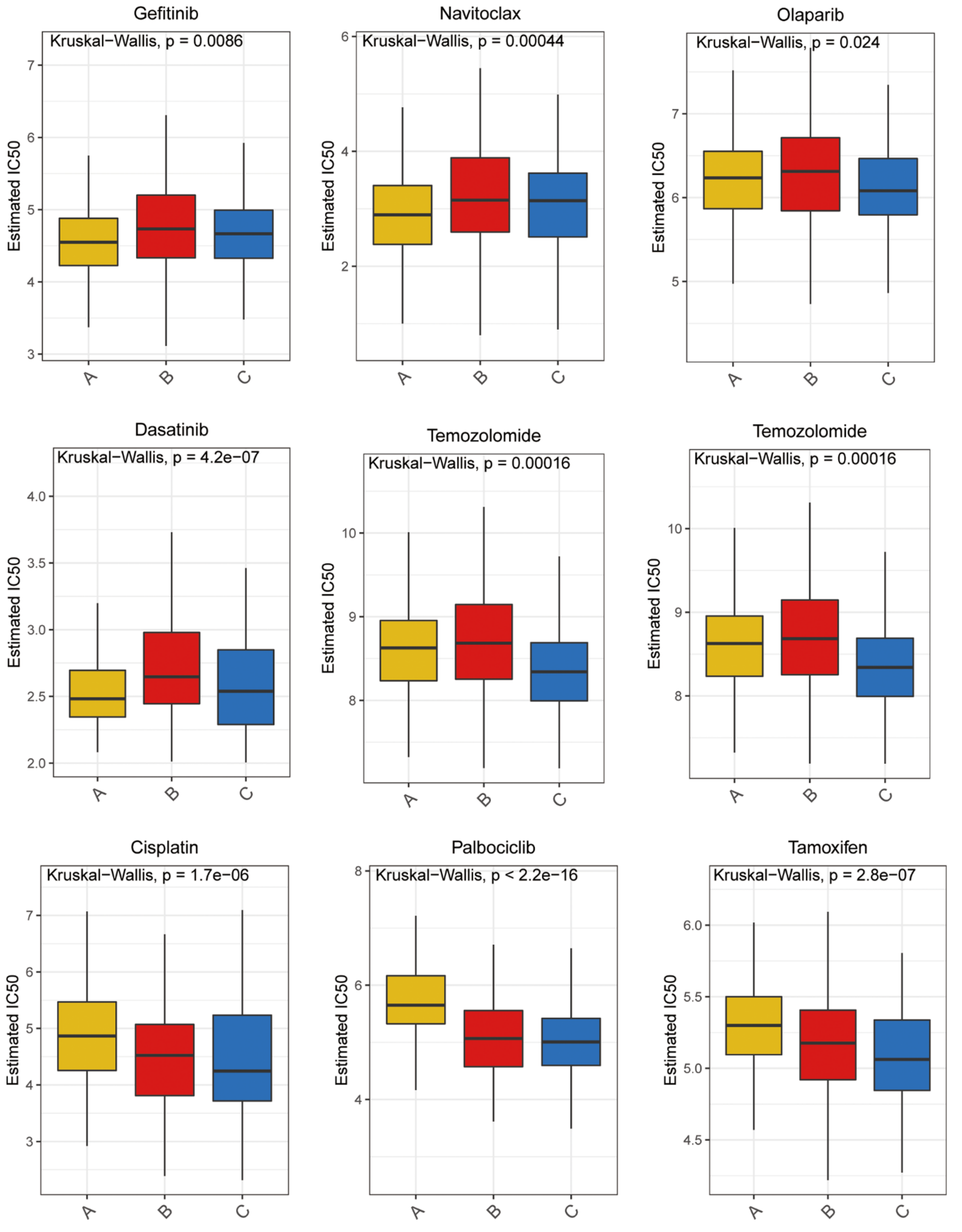

Supplementary Figure 5. Comparison of drug sensitivity. Estimated IC50 of the indicated molecular targeted drugs in Cluster A, B, and C.

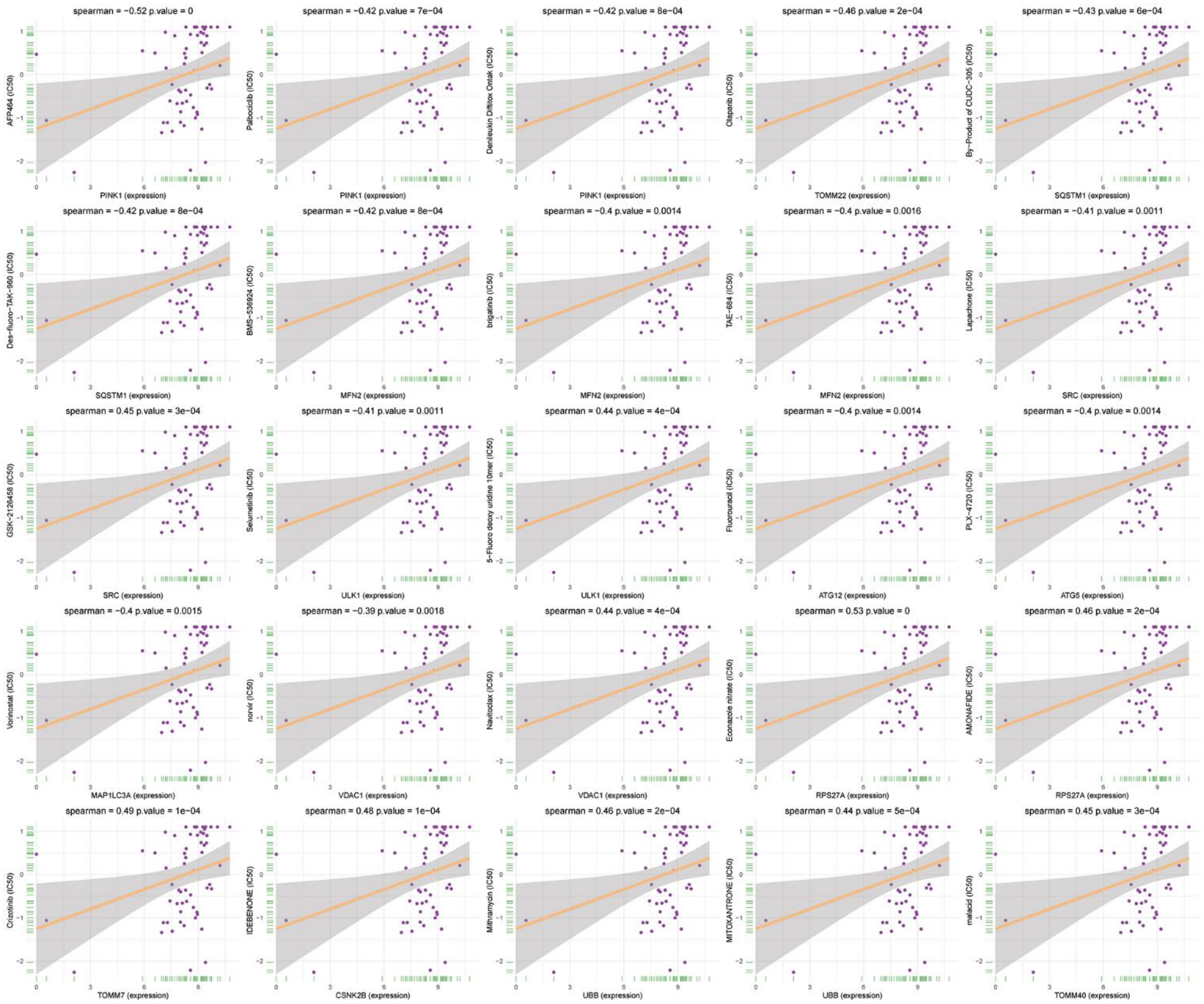

Supplementary Figure 6. The association between mitophagy gene expression and drug sensitivity based on the CellMiner database.

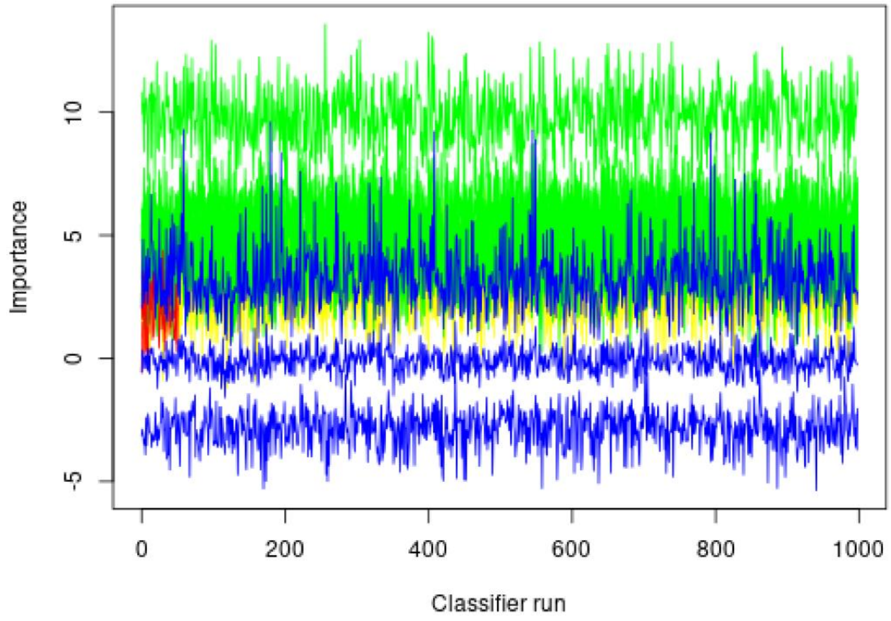

Supplementary Figure 7. Results of the Boruta algorithm iterations. Green indicates features considered important by the Boruta algorithm while blue represents shadow attributes.

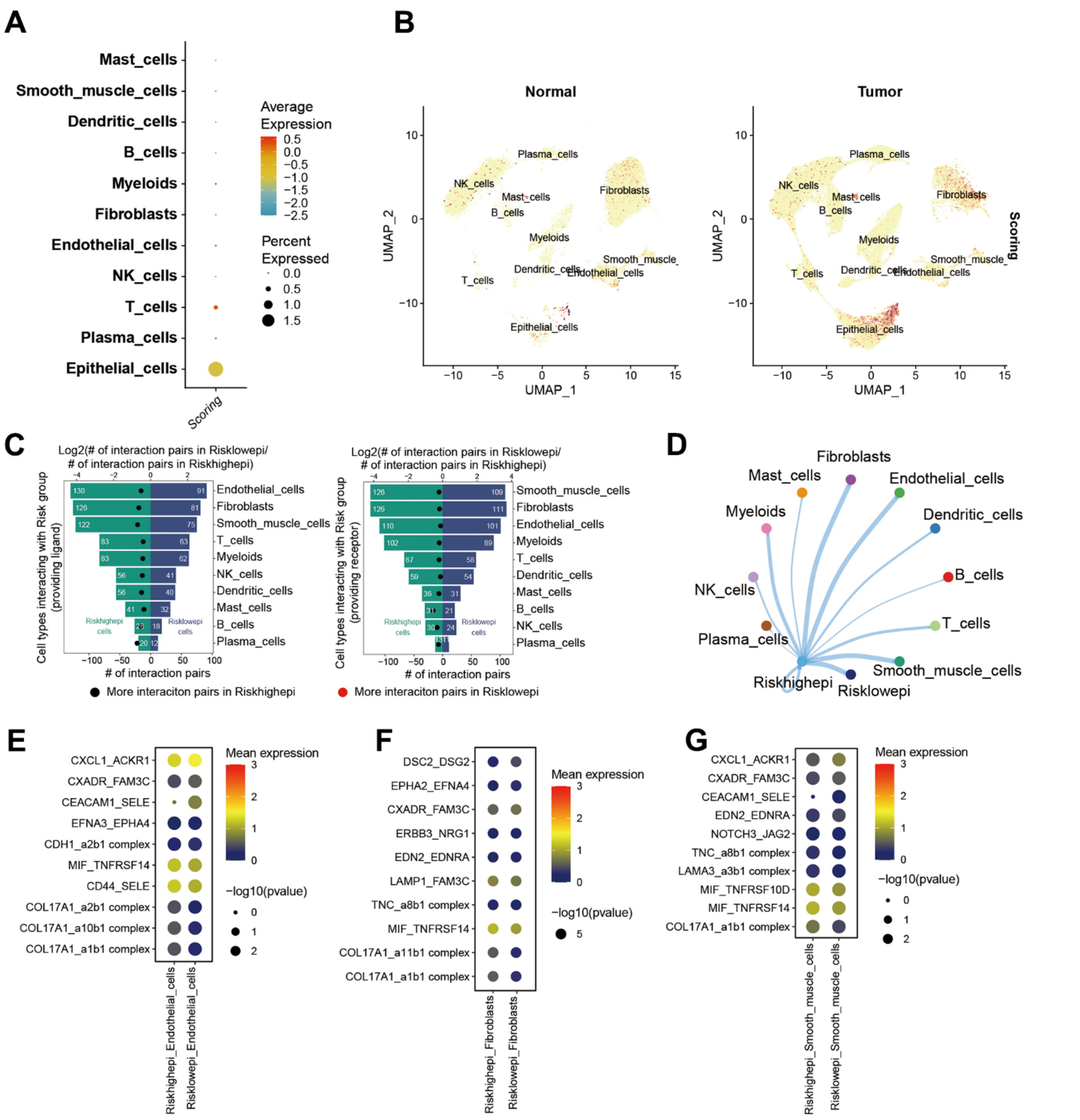

Supplementary Figure 8. Observations on cell-to- cell communication. (**A**) Scores for each cell type shown in a dotplot. (**B**) Dimensionality reduction charts show the risk scores of different cell types, grouped by normal and tumor. (**C**) Overview of cellular communication. (**D**) Network visualization of high-risk epigenetic profiles' communication patterns. (**E**–**G**) Bubble plots depicting communication intensity between various cell types and the Risk group. This includes interactions from the Risk group to Endothelial cells (**E**), Fibroblasts (**F**), and Smooth muscle cells (**G**) to the Risk group.

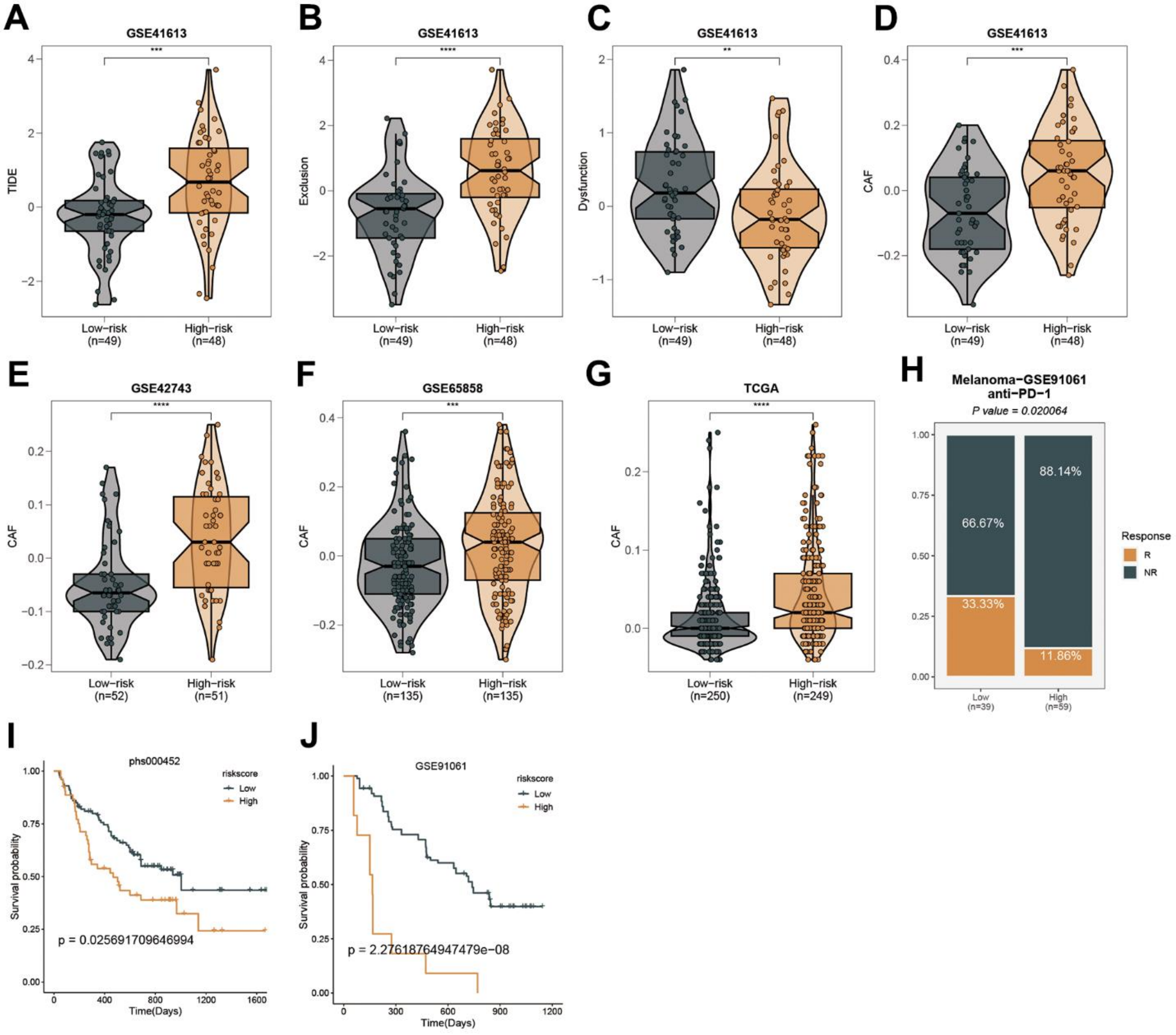

Supplementary Figure 9. Predicting immune therapy response with MSRS. (**A**–**C**) Comparison of the TIDE (**A**), dysfunction (**B**), and exclusion (**C**) between groups with high and low MSRS. (**D**–**G**) Comparison of CAF in high and low MSRS groups in GSE41613 (**D**), GSE42743 (**E**), GSE65858 (**F**), and TCGA (**G**). (**H**) A comparison of the MSRS between immune therapy responders and nonresponders in the GSE91061 immunotherapy cohort. (**I**, **J**) Based on the phs000452 (**I**), and GSE91061 (**J**) immunotherapy cohorts, the effect of MSRS on prognosis in these patients.

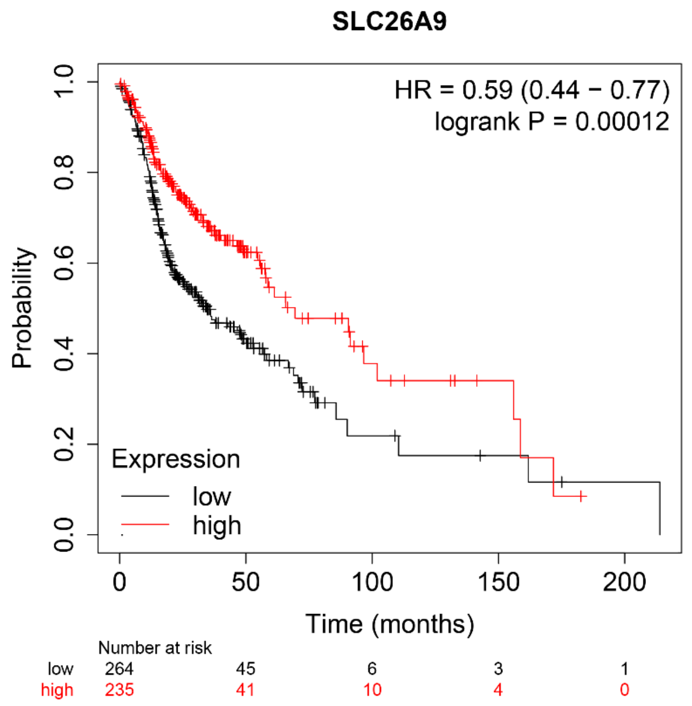

Supplementary Figure 10. KM survival analysis of SLC26A9.
