# Supplementary material for: Multi omics analysis of mitophagy subtypes and integration of machine learning for predicting immunotherapy responses in head and neck squamous cell carcinoma
Source: Aging (Albany NY). 2024 Jun 21;16(12):10579–614. doi: 10.18632/aging.205964 (PMC11236326; doi:10.18632/aging.205964)
Supplement: Supplementary Tables 1 and 2 [file aging-16-205964-s002.pdf]

SUPPLEMENTARY TABLES

Supplementary Table 1. The specific transcription factors for HNSCC.

regulon
CSDE1
ZNF207
LTF
YBX1
PITX1
TP63
NFE2L1
XBP1
HIF1A
KLF5
TSC22D1
MAZ
USF2
STAT1
EPAS1
NFE2L2
SFPQ
IRF6
EGR1
ID1
ATF4
BHLHE40
MYC
HMGA1
SSRP1
ARID5B
ETS2
ZNF385A
HMGB2
PHB
STAT3
CNBP
FOS
JUNB
FOSL1
JUN
MAF1
ZFP36L1
LITAF
HMGB1
SREBF2
ATF6B
NME2

ZEB2
EBF1
FOXP3
TP53

Supplementary Table 2. The list of Mitophagy-Related Genes.

ATG12
ATG5
CSNK2A1
CSNK2A2
CSNK2B
FUNDC1
MAP1LC3A
MAP1LC3B
MFN1
MFN2
MTERF3
PGAM5
PINK1
PRKN
RPS27A
SQSTM1
SRC
TOMM20
TOMM22
TOMM40
TOMM5
TOMM6
TOMM7
TOMM70
UBA52
UBB
UBC
ULK1
VDAC1
