# Supplementary material for: Multi omics analysis of mitophagy subtypes and integration of machine learning for predicting immunotherapy responses in head and neck squamous cell carcinoma
Source: Aging (Albany NY). 2024 Jun 21;16(12):10579–614. doi: 10.18632/aging.205964 (PMC11236326; doi:10.18632/aging.205964)
Supplement: Supplementary Table 5 [file aging-16-205964-s005.pdf]

Supplementary Table 5. The result of Bootstrapping-based analysis.

	gene	times
1	GRHL3	1000
2	MS4A2	997
3	SCNN1B	996
4	ATP10B	995
5	A2ML1	992
6	TGM5	981
7	BNIPL	978
8	SASH1	977
9	ALOX12B	965
10	KRT80	893
11	PLA2G3	891
12	SPINK5	867
13	ZNF831	781
14	SLC26A9	749
15	LY6G6C	742
16	MASP1	722
17	MUC15	683
18	TMPRSS11D	675
19	FAM83C	649
